# Validation of self-hearing test in non-clinical setting: a systematic literature review

**DOI:** 10.1016/j.bjorl.2026.101803

**Published:** 2026-04-16

**Authors:** Nor Hidayah Mohammed Hatta, Wan Najibah Wan Mohamad, Mohd Normani Zakaria, Mohd Fadzil Nor Rashid, Mohd Syaiful Nizam Abu Hassan, Hasrul Hosshan

**Affiliations:** a Universiti Sains Malaysia, School of Health Sciences, Audiology Programme, Health Campus, Kelantan, Malaysia; b Hospital Tuanku Ja'afar Seremban, Otorhinolaryngology Department, Negeri Sembilan, Malaysia; c Universiti Sultan Zainal Abidin, Terengganu, Malaysia; d Sultan Idris Education University, Faculty of Human Development, Centre for Inclusive Research on Community and Disability (CIRCLE), Perak, Malaysia

**Keywords:** Self-hearing tests, Validation, Nonclinical, Pure tone audiometry, Mobile hearing test

## Abstract

**Objectives:**

This systematic literature review is focused on comparing automated audiometry with manual audiometry in nonclinical settings, with the primary objective of obtaining a comprehensive understanding of the advantages, limitations, and overall efficacy of automated audiometry.

**Methods:**

Following the Preferred Reporting Items for Systematic Reviews and Meta-Analyses (PRISMA 2020) guidelines and being registered at PROSPERO, the review utilises Joanna Briggs Institute Critical Appraisal tools to evaluate the quality and rigour of 11 selected research studies.

**Results:**

Key findings highlight sensitivity and specificity ranging from 71% to 98.5%. Time efficiency considerations reveal a 12.3% faster smartphone screening time than conventional screening. The validation in children, as demonstrated, underscores the potential use of self-administered applications as initial tests for hearing problems in this demographic.

**Conclusion:**

This comprehensive review underscores the validity, reliability, and potential advantages of automated audiometry over traditional methods in nonclinical settings. While manual audiometry has long been considered the gold standard, automated audiometry demonstrates comparable outcomes, yielding clinically acceptable audiograms and offering potential cost-saving benefits.

## Highlights

• Automated audiometry achieves 71%–98.5% accuracy in nonclinical settings.
• Smartphone hearing screenings are 12.3% faster than conventional methods.
• Automated self-hearing tests are promising for early detection in children.
• Automated audiometry delivers reliable audiograms and reducing costs.
• Automated audiometry improves hearing healthcare access, valid and reliable.

## Introduction

Recent key figures from the World Health Organization (WHO) indicate that more than 5% of the global population, equivalent to 430 million people, requires rehabilitation for disabling hearing loss. By 2050, it is projected that over 700 million individuals, or 1 in every ten people, will experience disabling hearing loss.^1^ Hearing loss, categorised as mild, moderate, severe, or profound, is defined by an individual's inability to listen as well as someone with normal hearing.^2^

Hearing healthcare involves subjective and objective assessments, with subjective tests requiring patient responses to stimuli and objective tests not relying on patient response.^3^ The most common subjective test is Pure-Tone Audiometry (PTA), which assesses whether hearing acuity is normal or impaired.^4^ PTA, measuring air and bone conduction hearing thresholds at specific frequencies, is considered the gold standard for hearing diagnosis.^5^

The evolution of audiometry began 150 years ago with tuning fork tests, progressing to the development of pure-tone audiometry in the 19th century and, in 1947, George Von Bekesy's invention of self-recording audiometry.^6^ The subsequent decades witnessed sophisticated attempts at automation, addressing the need for large-scale hearing loss screening.^6^

Globally, the increasing number of individuals with hearing loss surpasses the availability of audiologists providing hearing healthcare services.^7^ With 86% of respondents indicating a shortage of audiologists in 64 surveyed countries, the need for alternative approaches, such as automated pure-tone audiometry, becomes apparent.^8^^,^^9^ As unrealistic as it is to expect a substantial increase in audiologist numbers, the hope lies in alternative approaches.^10^ Margolis and Morgan (2008) emphasised the potential of automated audiometry to increase access, optimise professional time, and reduce costs. Self-assessment technologies, including automated hearing assessment tools, address the shortage of audiology professionals.^11^

While automated audiometry has existed for years, its widespread use for diagnostic purposes has been limited. It primarily focuses on screening tests and tracking methods, excluding bone-conduction testing and contralateral masking due to added complexity.^9^ The increasing demand for hearing services in overcrowded clinics and remote areas, coupled with challenges in training more audiologists and issues related to remote living, highlights the problem of under-servicing.^8^ Margolis and Morgan (2008) estimate a substantial gap between needed and available audiograms in the United States alone, emphasising the urgency of exploring alternative solutions.^11^

The well-defined protocol for pure-tone audiometry logically lends itself to automation.^9^ Though automated audiometry cannot replace an audiologist, its potential to determine pure-tone thresholds with accuracy comparable to manual audiometry is a valuable asset in meeting the demand for hearing health services.^11^ Advances in technology now make automating diagnostic pure-tone audiometry feasible, utilising PC-based or microprocessor-driven modern audiometers that implement well-established testing systems. Despite automated testing facing criticism for its inability to replace highly trained professionals fully, it offers potential benefits in terms of efficiency, validation, reliability, usability and cost-effectiveness.^9^^,^^12^

Shojaeemend's (2018) review supports the clinically acceptable results of automated audiometry compared to traditional methods, emphasising its cost-saving and accessibility advantages. However, environmental noise impact and quality assurance challenges require further investigation.^13^ The historical progression from early electronic audiometers to the current state of audiometry underscores the need for continued exploration and validation of automated methods.^14^, ^15^, ^16^ With audiology transitioning into a doctoral-level profession, the value lies not in routine testing but in result interpretation and implementing rehabilitative strategies.^9^

Automated audiometry can increase access, standardise test procedures, and decrease test administration costs.^9^ Providing valid results enables audiologists to focus on result interpretation, referral decisions, and management planning, especially in teleaudiology in nonclinical settings, such as in schools.^17^^,^^18^

This systematic literature review is centred on self-hearing tests to contrast automated audiometry with manual audiometry in a nonclinical setting. The main aim is to understand automated audiometry's advantages, limitations, and overall efficacy. Through this systematic comparison, we seek to address the following queries: Is the validity of the automated hearing threshold suitable for implementation in a non-clinical setting?

## Methods

This systematic review was conducted according to the Preferred Reporting Items for Systematic Reviews and Meta-Analyses (PRISMA 2020) guidelines. Our review is also registered at PROSPERO under registration number CRDXXXXX.

### Search strategy

The search strategy involved a systematic exploration of relevant articles conducted by two independent researchers on three separate occasions: 20 August 2023, 21 August 2023, and subsequently on 24 August 2023. The electronic databases employed for this systematic search included Web of Science, PubMed, SCOPUS, and the Cochrane Central Register of Controlled Trials (CENTRAL). Utilising the specified databases, we conducted a search using identified keywords and index terms across all included databases. Additionally, we searched the reference lists of all identified reports and articles for additional studies.

The studies were identified using the following terms and Boolean operators: ("automated audiometry" OR "automated hearing test" OR "self-hearing test" OR "self-test audiometry" OR "self-audiometry test") AND ("validation" OR "verification" OR "agreement"). The publication year range considered in the search was between 1990 to 2023.

### Inclusion and exclusion criteria

To ensure the adherence of selected articles to our inclusion criteria, two researchers (referred to as Author 1 and Author 2) independently screened the title, abstract, and full text of records identified from the search. Simultaneously, the remaining two authors independently reviewed all the articles selected during the initial search. Our systematic review included studies that met the following criteria: Firstly, studies focusing on self-hearing tests or automated audiometry were considered. Secondly, studies conducted in nonclinical settings were included. Finally, studies that validated self-hearing audiometry tests compared to manual audiometry were also incorporated.

Additionally, our focus was on observational cross-sectional studies, and we exclusively included studies published in English. We excluded qualitative studies, letters to the editor, proceedings, review articles, animal studies, and individual case reports from our analysis to maintain a clear scope and relevance.

In instances of disagreements or discrepancies in the selection process, the four authors engaged in constructive discussions to reach a consensus and resolve any differences. All four authors adhered to the latest PRISMA 2020 guidelines as the standard protocol for the search process. The aim was to ensure a rigorous and transparent selection process, following the PRISMA 2020 guidelines and maintaining the quality and reliability of the review's findings. Duplicate records in the search results were removed using Mendeley Reference Manager. After the eligibility assessment of reports, we included studies conducted in a nonclinical setting, in contrast to those performed in a clinical or laboratory setting.

### Participants/Population

The criteria for participants include both adults and school-age children, encompassing both normal-hearing and hearing-impaired populations. The key aspect of interest is hearing sensitivity, measured in Decibels (dB). Studies will be considered using various methods, such as Hughson-Westlake, machine-based, Bekesy, or other threshold-seeking approaches.

Participants with any visible, active ear infection will be excluded from the review to maintain relevance and precision. This exclusion criterion ensures that the study focuses on individuals without concurrent ear infections, thus allowing a more accurate assessment of hearing sensitivity using the specified methods.

### Main outcomes

The primary objective of this review is to evaluate the feasibility of implementing self-hearing tests in nonclinical settings. The review focuses on assessing the validation, test-retest reliability, and testing duration of self-hearing tests compared to manual hearing tests. In investigating the validation of self-hearing tests, the review aims to determine the accuracy of these tests in identifying hearing impairments and distinguishing them from normal hearing conditions. Additionally, the review assesses the test-retest reliability of self-hearing tests to comprehend the consistency of results obtained through repeated testing. This reliability analysis is crucial in understanding the stability of self-hearing test outcomes over time, contributing to the overall reliability of these tests. Moreover, the review compares the testing duration of self-hearing tests, shedding light on their time efficiency. Understanding the time required for these tests is essential for their practical implementation in nonclinical settings.

## Results

### Identification and selection of studies

The systematic search identified 378 potentially relevant citations, with 303 articles from Web of Science, 38 from the Cochrane Library, 22 from PubMed, and 15 from Scopus. The studies found in the database were further organised using the Mendeley reference manager. Using the “Find duplicates” function, 45 duplicate records were removed. Subsequently, the remaining 343 records underwent further screening based on titles and keywords, resulting in the exclusion of 285 records that did not fulfil the inclusion criteria of this review. After the initial screening, 58 records remained.

A second screening was conducted, leading to the exclusion of 17 records for specified reasons. The remaining 41 records underwent a thorough review, resulting in the exclusion of 30 records that were conducted in a clinical setting and did not fulfil the inclusion criteria for our review. The remaining 11 records were further assessed using Critical Appraisal Tools for JBI Systematic Reviews, with details outlined in the methodological quality check.

Following the database screening process, other methods initially identified nine records. However, all nine were excluded as they did not meet the inclusion criteria. Ultimately, only 11 full-text articles were included in this systematic review. Appendix 1 illustrates the PRISMA flow chart depicting our study's identification and selection process.

### Critical appraisal

In this study, we employed the Joanna Briggs Institute Critical Appraisal tools to evaluate the quality and rigour of the included research studies. Using these appraisal tools enables us to identify potential biases, limitations, and strengths in the included studies, aiding in the more effective interpretation of evidence and formulating informed conclusions and recommendations based on the best available evidence. For instance, a reviewer may find that in Study 1, there was a low risk of bias for the domain “selection and allocation”, as all questions received a response of “yes”. Conversely, for Study 2, a reviewer may identify a moderate risk of bias for the same domain, as one of the questions received a “no” response.

As suggested by Dol and colleagues, the methodological quality was set with a 50% cutoff, as per the protocol, and any study scoring below 50% was excluded from this review.^16^ Any disagreements among reviewers were resolved through discussion. Table 1 presents the study results following critical appraisal using the revised JBI critical appraisal tool checklist for analytical cross-sectional studies.

**Table 1:** Presentation of results following critical appraisal using the revised JBI critical appraisal tool for analytical cross-sectional studies.

Authors, Year	Q1	Q2	Q3	Q4	Q5	Q6	Q7	Q8	%	Overall appraisal
Honeth et al. (2010)^19^	Yes	Yes	Yes	Yes	Yes	Yes	Yes	Yes	100	Include
Eikelboom et al. (2013)^20^	Yes	Yes	Yes	Yes	Yes	Yes	Yes	Yes	100	Include
Mahomed-Asmail et al. (2016)^21^	Yes	Yes	Yes	Yes	Yes	Yes	Yes	Yes	100	Include
Margolis et al. (2016)^22^	Yes	Yes	Yes	Yes	Yes	Yes	Yes	Yes	100	Include
Masalski et al. (2016)^23^	Yes	Yes	Yes	Yes	Yes	Yes	Yes	Yes	100	Include
Whitton et al. (2016)^24^	Unclear	Yes	Yes	Yes	Yes	No	Yes	Yes	75	Include
Govender & Mars (2018)^25^	Unclear	Yes	Yes	Yes	Yes	Yes	Yes	Yes	87.5	Include
Chu et al. (2019)^26^	Yes	Yes	Yes	Yes	Yes	Yes	Yes	Yes	100	Include
Mosley et al. (2019)^27^	Yes	Yes	Yes	Yes	Yes	Yes	Yes	Yes	100	Include
Mealings et al. (2020)^28^	Yes	Yes	Yes	Yes	Yes	Unclear	Yes	Yes	87.5	Include
Hopping & Krishnan (2022)^29^	Yes	Yes	Yes	Yes	Yes	Yes	Yes	Yes	100	Include

Q1: Were the criteria for inclusion in the sample clearly defined?; Q2: Were the study subjects and the setting described in detail?; Q3: Was the exposure measured in a valid reliable way?; Q4: Were objective, standard criteria used for measurement of the condition?; Q5: Were confounding factors identified?; Q6: Were strategies to deal with confounding factors stated?; Q7: Were the outcomes measured in a valid and reliable way?; Q8: Was appropriate statistical analysis used?

### Data extraction

Two independent reviewers performed data extraction from all included studies. In case of disagreements during the extraction process, a third reviewer was consulted to facilitate discussion and reach a consensus. Only study findings that met the predefined inclusion criteria were extracted, ensuring relevance and alignment with the systematic review's objectives. This meticulous data extraction process aimed to capture comprehensive details from each study, contributing to the robustness and reliability of the review's findings. Details of the above-mentioned 11 articles are presented in Table 2.

**Table 2:** Main results of the studies including intervention/comparator, study subject/setting and research tool used.

Author / Year	Intervention/ Comparator	Study Subject	Testing Setting	Automated Research Tool	Outcome/Result	Country
Chu et al., 2019^26^	Air conduction pure-tone screening and PTA average	85 school-age children aged 11–12 y/o	Elementary school	iOS-based automated Ear Scale app (version 2.0)	Sensitivity and specificity: 100%	Taiwan
Eikelboom et al., 2013^20^	Test-retest reliability and validation of AMTAS	44 adults, 30–80 years	Quiet room	AMTAS (Automated Method in Testing Auditory Sensitivity)	Hearing threshold differences: 0.1 dB for air conduction, and 0 dB for bone conduction.	Australia
Govender & Mars, 2018^25^	Validating automated air and bone conduction testing.	50 students, aged 6–13 y/o, with normal and sensorineural hearing loss.	Noise-controlled school environment	KuduWave 5000	No significant hearing threshold difference at 250–2000 Hz and 8000 Hz.	South Africa
Honeth et al., 2010^19^	Comparison: Pure tone automated air conduction average vs PTA average.	72 participants, aged 19–71 y/o, with a mean age of 45	Ordinary office at the clinic	JAVA program (self-administered Internet-based hearing test)	Sensitivity: 75% Specificity: 96% Test-retest (ICC): 0.99	Sweden
Hopping & Krishnan, 2022^29^	Automated game-based and conventional approach	58 children aged 5–12 y/o	After-school facilities	Apple® iPad with SHOEBOX Audiometry Standard Edition Software	±5 dB difference: 85% ±10 dB difference: 8% >±10 dB difference: 6.25%	USA
Mahomed-Asmail et al., 2016^21^	Validity of a hearing screening using smartphone	1070 school-age children 5–12 y/o.	Quiet room at school	Samsung Galaxy S5301 phones for the hearScreen™	Sensitivity: 75% Specificity: 98.5% Testing time: 12.3% faster	South Africa
Margolis et al., 2016^22^	Air-conduction audiogram at ﬁve test frequencies	28 participants, aged 44–88 y/o	Home	The Home Hearing Test (HHT)	Threshold differences: ±5 dB difference: 71% ±10 dB difference: 91% ±15 dB difference: 96%	USA
Masalski et al., 2016^23^	Comparing reference sound level and estimated hearing threshold.	79 adults in the control group	Home for uncontrolled group	Free mobile phone app “Hearing Test”, available on Google Play	Difference in sound level between control and uncontrolled group: 1.50 dB	Poland
Mealings et al., 2020^28^	Comparison between clinician-administered and self-administered hearing tests	297 Aboriginal and Torres Strait Islander children aged 4–14 y/o	Quiet rooms at schools	AutoAud and Sound Scouts	AutoAud, ±5 dB: 88% Sound Scouts (>20 dB), sensitivity was 41% and specificity was 89%	Australia
Mosley et al., 2019^27^	Comparing air conduction hearing threshold for automated and manual testing	112 English-speaking adults, aged 60 y/o and older	Home	Etymotic Home Hearing Test (HHT)	±5 dB difference: 89.1% ±10 dB difference: 96.9% ±15 dB difference: 98.4%	USA
Whitton et al., 2016 ^31^	Comparing self-administered hearing threshold and conventional	40 adults with hearing-related concerns and non-hearing-related issues.	Home	Automated tablet audiogram application	The mean difference within the clinical equivalency margin from 500 Hz to 8000 Hz and good test-retest reliability	USA

The categorisation of these 11 studies is divided according to PICOS or the participants involved, type of intervention, type of comparison group, outcomes of interest, and study design. The population in this review comprises adults and school-age children, including both normal and hearing-impaired populations. The intervention refers to the hearing thresholds determined by at least, the Hughson-Westlake technique, machine, Bekesy and another threshold-seeking method. For the comparator/comparison group, we will compare automated and conventional manual audiometry. Outcome interests will include validation (reliability and time efficiency) and automated audiometry in a nonclinical. Lastly, the study design is a cross-sectional study using English journals.

### Sensitivity and specificity

Three studies in this review show outstanding sensitivity and specificity.^26^^,^^19^^,^^21^ Chu et al. (2019) showed 100% sensitivity and specificity while Honeth et al. (2010) showed 75% sensitivity and 96% specificity.^26^^,^^19^ Honeth also reported excellent test-retest reproducibility, with a Pearson correlation coefficient of 0.99 (p < 0.0001) for both ears.^19^ Another study reported no statistically significant difference in performance between conventional and smartphone hearing screening techniques, demonstrating equivalent sensitivity (75.0%) and specificity (98.5%).^21^

In a study by Eikelboom and colleagues, test-retest reliability for the overall difference in air conduction hearing thresholds for self-hearing tests (n = 119) was reported at 0.5 dB.^20^ For accuracy, the overall difference in air conduction hearing thresholds (n = 509) between the self-hearing and conventional hearing tests was 0.1 dB. They conclude that variations between air and bone conduction audiometry for automated and manual audiometry were within normally accepted limits for audiometry.

Correlation analyses done by Mosley et al. (2019) revealed signiﬁcant frequency-speciﬁc correlations, ranging from 0.91 to 0.97 (p = 0.001), for air-conduction thresholds between self-hearing test and manual audiometry.^27^ Mean self-hearing test thresholds were signiﬁcantly correlated with mean manual audiometry thresholds in both ears across the frequency range. This relationship is held across different degrees of hearing loss. Whitton et al. (2016) also reported similar findings.^31^ In this study, they reported that self-hearing test audiograms were statistically equivalent to manual, clinic-based testing audiograms (p ≤ 0.02). They also concluded that these data prove that several self-administered, automated hearing measurements are statistically equivalent to manual measurements. The demonstration of statistical equivalency for these basic behavioural hearing tests points toward the eventual feasibility of monitoring progressive or fluctuating hearing disorders outside the clinic to increase the efficiency of the collected clinical information.

### Time efficiency

Time efficiency was also an element reported in self-hearing tests. A study reported that Smartphone screening (hearScreen™) was 12.3% faster than conventional screening.^21^ On the downside, a study by Hopping and Krishnan (2022) on 58 children found that the currently available automated game-based procedure takes longer than the tester-assisted mode.^29^ They concluded that although tablet audiometry is accurate and reliable, the presently available automated game-based procedure takes longer than the tester-assisted mode.

The ability of a self-hearing test to provide accurate results raises the prospect of optimising the efficiency of audiologists, allowing audiologists to allocate more time to other crucial aspects of hearing health care. This could lead to improved patient access to hearing services, particularly in areas facing a shortage of audiologists and hearing healthcare professionals. However, despite the promising features of self-hearing tests, certain challenges require further exploration. Environmental noise can potentially impact the accuracy of test results, necessitating robust noise monitoring measures to ensure reliable outcomes. Ultimately, this review underscores the transformative potential of self-hearing tests in enhancing hearing health services globally and addressing the needs of underserved populations. One self-hearing test tool that has caught our attention and that we intend to explore further its relative merits is AMTAS.

### Validation of self-hearing test in children

Five of the eleven studies in this review involved validation of self-hearing tests among children. Mealings et al. (2020) show the potential of using self-administered applications as initial tests for hearing problems in children.^28^ Self-administered tests allow all children to be tested for hearing problems, and then those who show issues can be referred for a more complete assessment.

Mahomed-Asmail et al. (2016) and Chu et al. (2019) both showed excellent sensitivity and specificity.^21^^,^^26^ They advocated that smartphone hearing screening offers an inexpensive alternative to conventional screening audiometry with specific application to school-based screening.^21^ The application utilises inexpensive, widely available smartphone and headphone technology for hearing screening. Sensitivity was similar and specificity was slightly better for the smartphone hearing screening device than conventional audiometry. Govender and Mars (2018) significantly correlated air and bone conduction thresholds.^25^ They reported that air and bone conduction thresholds for both methods were within 5 dB agreement of each other for over 80% of normal-hearing ears.

### Bone conduction and interaural attenuation

In the studies by Govender and Mars (2018) and Eikelboom et al. (2013), Bone Conduction (BC) thresholds were obtained using a Radioear B-71 bone oscillator positioned on the forehead, with participants’ ears covered by headphones or insert earphones.^25^^,^^20^ In Govender and Mars (2018), the KUDUwave 5000 automated audiometer was used, applying the modified Hughson-Westlake method for threshold determination.^25^ Masking during BC testing was automatically presented at 20 dB above the air-conduction threshold. The authors acknowledged possible variability arising from oscillator placement, contact pressure, calibration inconsistencies, and vibrotactile responses, particularly in cases of asymmetrical or severe hearing loss where masking can be less reliable.

Similarly, Eikelboom et al. (2013) employed automated BC audiometry with the oscillator placed on the forehead and participant-guided on-screen instructions.^20^ The study emphasized that BC testing tends to exhibit greater variability than air conduction due to the complex transmission pathways of bone conduction, the influence of transducer placement and coupling force, and vibrotactile perception at low frequencies. Although neither study described distinct procedures for managing asymmetrical losses beyond standard masking, both recognized that such conditions could introduce additional variability and affect the accuracy and reliability of automated BC thresholds.

## Discussion

### Advantages of self-hearing test

In our systematic review, the analysis of various self-hearing tests in a nonclinical setting revealed several advantages of automated audiometry. Automated audiometry demonstrated comparable outcomes to traditional manual audiometry methods, often considered the gold standard. Studies such as Honeth et al. (2010) and Chu et al. (2019) showcased outstanding sensitivity and specificity, with potential cost-saving benefits.^19^^,^^26^ Mahomed-Asmail et al. (2016) also reported equivalent sensitivity and specificity between conventional and smartphone hearing screening techniques.^21^ Eikelboom et al. (2013) found test-retest reliability within accepted limits, further supporting the feasibility of automated audiometry.^20^ Whitton et al. (2016) and Mosley et al. (2019) demonstrated significant correlations and statistical equivalency between self-hearing tests and manual audiometry, emphasising their reliability and accuracy.^24^^,^^27^

The validity of a screening protocol is determined by the degree to which results are consistent with the actual presence or absence of the disorder.^29^ Sensitivity and specificity values support the validity of a screening technique. Sensitivity indicates the accuracy of the screening tool in correctly identifying individuals with the target condition, while specificity refers to the accuracy of the screening tool in correctly identifying individuals without the target condition.^30^, ^31^, ^32^

Furthermore, time efficiency was highlighted in studies such as Mahomed-Asmail et al. (2016), reporting faster screening with smartphone methods.^21^ Although Hopping and Krishnan (2022) found that automated game-based procedures took longer in certain cases, the overall potential for time savings in self-hearing tests was evident.^29^ The ability of self-hearing tests to yield accurate results could optimise audiologists' efficiency, potentially improving patient access to hearing services, particularly in regions with a shortage of healthcare professionals.

Another main advantage of automated audiometry is saving costs and improving accessibility to hearing care, which can lead to a cost-effective and rapid diagnosis of hearing impairment, especially in poor areas. Research done by Margolis and Morgan in 2008, analysing the capacity, need and benefit of automated pure-tone audiometry, concluded that automation would and should be utilised to increase access to hearing services, allow better utilisation of professional time, and decrease the cost of basic tests. The use of self-assessment technologies, such as the development of automated hearing assessment tools, is one way to address the shortage of audiology professionals.^11^

### Limitations of automated audiometry

While automated audiometry presents several advantages, certain limitations require consideration in ensuring the quality of the automated audiometry test results. Environmental noise emerged as a potential factor affecting accuracy, emphasising the need for robust noise monitoring measures to ensure reliable outcomes. Govender and Mars (2018) emphasised the relevance of response time in children and the need for ongoing motivation and encouragement during automated testing.^25^ Hopping and Krishnan (2022) noted the efficiency of tester-assisted procedures compared to automated game-based procedures, highlighting areas for improvement to enhance engagement, particularly in pediatric settings.^29^

Govender and Mars (2018) also reported that concerning automated testing, the correct set-up of the patient must be prioritised.^25^ Careful insertion of the insert foam tips is integral to the success of automated testing. Several key clinical considerations emerged that could improve automated test results' reliability. These factors must be considered when developing protocols and guidelines for automated testing of children.

### Overall efficacy of self-hearing tests

The overall efficacy of self-hearing tests, as demonstrated by studies on AMTAS (Automated Method of Testing Auditory Sensitivity), appears promising. Developed in 2002 by Margolis, AMTAS has undergone extensive research and development, with studies confirming its validity. Eikelboom et al. (2013) and Margolis et al. (2016) reported variations within acceptable thresholds compared to manual audiometry.^20^^,^^22^ However, challenges related to background noise during testing and ensuring consistent hearing profiles must be addressed for accurate results. In most studies, the results of automated self-hearing tests were similar to the traditional approach, and it seems that traditional audiometry can be replaced with an automated approach.

## Conclusion

Self-hearing tests offer a promising avenue for transforming hearing health services, especially in meeting the growing need for accessible testing in urban clinics and remote regions lacking adequate resources. This review focuses on the validity and utility of automated audiometry, comparing it to traditional manual methods. While manual audiometry has long been the benchmark, automated audiometry demonstrates comparable sensitivity and specificity, providing clinically acceptable audiograms and potential cost-saving benefits. The efficiency of automated audiometry suggests a potential for optimising audiologists' time, thus improving patient access to hearing services, particularly in regions with limited healthcare professionals. However, environmental noise impact requires further investigation and mitigation strategies.

## ORCID ID

Wan Najibah Wan Mohamad: 0000-0002-7640-1228

Mohd Fadzil Nor Rashid: 0000-0002-4020-7036

Mohd Syaiful Nizam Abu Hassan: 0000-0002-1612-2315

Hasrul Hosshan: 0000-0003-1948-8205

## CRediT authorship contribution statement

Conception and design: NHMH, MFNR. Analysis and interpretation of the data: NHMH, MFNR. Drafting of the article: NHMH, MFNR. Critical revision of the article for important intellectual content: NHMH, MFNR, HH. Final approval of the article: NHMH, WNWM, MNZ, HH, MFNR. Obtaining funding: MNFR, MSNAH, HH. Administrative, technical or logistic support: MNFR, MSNAH, HH. Collection and assembly of data: NHMH, MFNR.

## Data availability statement

The authors declare that all data are available in repository.

## Declaration of competing interest

The author(s) declared no potential conflicts of interest concerning this article's research, authorship, and/or publication.
