# Supplementary material for: Validation of self-hearing test in non-clinical setting: a systematic literature review
Source: Braz J Otorhinolaryngol. 2026 Apr 16;92(3):101803. doi: 10.1016/j.bjorl.2026.101803 (PMC13099441; doi:10.1016/j.bjorl.2026.101803)
Supplement: Supplementary file 1 [file mmc1.docx]

**BJORL-D-25-00028_Supplementary Material**

**Appendix 1** PRISMA chart of the study's identification and selection process.
